# Analysis of the relationship between urban dynamics and prevalence of remote work based on population data generated from cellular networks

**DOI:** 10.1038/s41598-023-47513-x

**Published:** 2023-11-17

**Authors:** Hiroto Akatsuka, Masashi Toyoda

**Affiliations:** 1 The University of Tokyo, Tokyo, Japan; 2 NTT DOCOMO, INC., Tokyo, Japan; 3 Institute of Industrial Science, The University of Tokyo, Tokyo, Japan

**Keywords:** Information technology, Statistics, Infectious diseases

## Abstract

The COVID-19 pandemic has accelerated the introduction of remote work as one way to ensure employee safety and reduce the amount of interpersonal contact while continuing business operations. Knowing the degree of prevalence of remote work and its establishment are considered to be important factors in future policies and urban planning. We applied non-negative matrix factorization to population-change data obtained from a mobile phone network to extract the component of working people, and analyzed the changes pre- and post-pandemic. Using the Wilcoxon signed-rank test, we confirmed that the number of people working in office and residential districts has significantly changed at a significance level of 1% in urban areas centered around Tokyo and Osaka, the two largest cities in Japan. Time-series data show that the number of workers is decreasing in office districts and increasing in residential districts, suggesting increased prevalence of remote work due to the COVID-19 pandemic and that it has become established. In addition, in urban areas centered around Tokyo, we confirmed that there is a moderate correlation between the changes in the number of people working in office districts and the capital size of corporations in the same area.

## Introduction

Countries around the world have implemented lockdowns and other non-pharmaceutical interventions to prevent the spread of coronavirus by reducing contact between people. In Japan, on February 27, 2020, a request to close schools was issued to local governments. On April 7th, a state of emergency was declared for seven prefectures, including Tokyo, and on April 16th it was expanded to include all of Japan. In declaring a state of emergency, then Prime Minister Abe called for people to “reduce contact with others by 70% to 80%” and “to work from home instead of at the office in principle”^1^. Unlike lockdowns in the United States and Europe, Japan’s declaration of a state of emergency does not have a legally binding effect and does not impose punishments such as fines or arrests on those who disregard the restrictions. However, many people refrained from going out^2^. Companies and other organizations have also been forced to change and this has accelerated the introduction of remote work as one way to ensure the safety of employees while continuing business activities^3^. Due to the declaration of a state of emergency, the remote work implementation rate in companies increased from 17.6% before the declaration to 56.4%, and the percentage of people who implemented remote work (for full-time employees) increased from 13.2% before the declaration to 27.9%^4^.

Remote work, also referred to as telework or telecommuting, refers to employees using Information and Communications Technology (ICT) to work from locations other than the office^3,5,6^. For many this means working from home, but it also encompasses working outside the traditional office. The concept of remote work is not new and has been around since the 1970s^6,7^. Remote work has developed in step with the development of ICT such as in terms of computers and the Internet^8^, but the COVID-19 pandemic has suddenly accelerated this trend.

Many studies have reported the benefits of remote work. Remote work generally increases productivity and job satisfaction^9–12^. The key to this is increased autonomy for employees, which provides more flexibility in choosing their working hours and facilitates finding a work-life balance^13^. In addition, from the perspective of companies and the environment, the promotion of remote work lower costs as it allows them to reduce office space^14^, maintain competitiveness in the competition to acquire human resources^15^, and reduce traffic volume and environmental pollution^16^. On the other hand, disadvantages to remote work have also been reported such as a decrease in satisfaction and productivity and an increase in working hours^8,17–19^. One of the reasons for these seemingly contradictory results lies in the ambivalence of remote work^20^. For example, remote work increases work flexibility, but simultaneously it increases a feeling of loneliness and weakens human networks within the company^21,22^. Especially in the case of the semi-forced transition to remote work due to the COVID-19 pandemic, it was a first-time experience for employees with almost no preparation time^23,24^, and in fact, not all of them wanted to transition to remote work. Therefore, it is clear that in order to continue to enjoy the benefits of remote work, it is necessary to focus on not only things that can be directly measured as outputs such as productivity, but also on maintaining employee motivation and happiness^25^. A study on the relationship between remote-work frequency and employee satisfaction reported that the employee satisfaction level was the highest when the frequency of remote work was less than 50%^26^.

In order to promote remote work practically, it is necessary not only to consider its advantages and disadvantages, but also to consider countermeasures against the impediments to its implementation. A study that organized 70 surveys on the actual situation of remote work during the COVID-19 pandemic in Japan showed that the reasons for not introducing or implementing remote work are greatly influenced by three factors^27^: “There are no jobs suitable for remote work,” “A remote work system has not been developed,” and “A remote work environment has not been developed.” Other obstacles include a lack of space for remote work at home from the employee perspective, a difficulty in evaluating performance from the company perspective, and high security risks^28,29^. In addition, the ease of implementing remote work is also affected by the culture of the country and organization^24,30^.

Knowing how prevalent remote work has become and, in the end, how established due to the COVID-19 pandemic is considered to be important to, for example, future city planning, infrastructure investment decisions, and disaster prevention plans. In this study, we use population data generated from operational data of a cellular network to confirm whether or not the number of people working in office/residential areas actually increased/decreased pre- and post-COVID-19 pandemic. Data from cellular networks have been used extensively in analyzing user behavior during the COVID-19 pandemic^2,31–33^. We use the real-time version of mobile spatial statistics (hereafter, RT-MSS), which represents population dynamics for the whole of Japan generated from the NTT DOCOMO cellular network.

In this investigation, we analyze the data from two viewpoints. The first is whether or not the number of people working in office/residential areas is significantly decreasing/increasing due to the prevalence of remote work related to the COVID-19 pandemic, and to what extent. The second is whether or not companies with larger capital are more likely to introduce remote work. Previous studies have shown that the higher the income level, the easier it is for employees to transition to remote work^32,34^. In this study, we focused on the corporate side and conducted a correlation analysis between the company capital size and the shift to remote work. We examined whether or not the introduction of remote work is progressing more smoothly in companies with large capital because it is easier to deal with environmental factors, which is one of the major obstacles.

We have used three datasets for our analysis. The first dataset is about the number of new COVID-19 positive cases in Japan, which is used for tracking the spread of the COVID-19 pandemic. The second dataset is RT-MSS, which we used to examine the prevalence of remote work during the COVID-19 pandemic by tracking population trends. Here, RT-MSS contains components related to people other than those working in the office, so it cannot be used as is for analyzing remote work trends. Therefore, we apply Non-Negative Matrix Factorization^35–37^ to decompose the population into various components and extract those related to working people. We then applied statistical tests to determine whether there were significant changes in the components related to working people in both office districts and residential districts in response to the COVID-19 pandemic. The third dataset is related to companies that have submitted securities reports. In Japan, stock-exchange listed companies are required to submit securities reports, and data related to these companies are publicly available. We use this data to analyze the impact of a company’s capital size on remote work by identifying areas where there are large companies, such as stock-exchange listed companies, and comparing them with areas where there are not.

## Materials and methods

### Number of new COVID-19 positive cases

**Figure 1:**
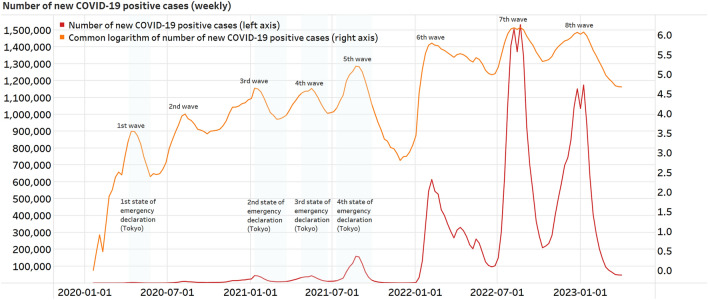
Number of new COVID-19 positive cases (weekly).

Regarding the situation of COVID-19 in Japan, we used daily data on the number of new COVID-19 positive cases provided by the Ministry of Health, Labor and Welfare ^38^. Figure 1 shows the weekly changes. In Japan, the infection began in late January 2020, and there have been eight confirmed waves of infection since then. The Japanese Ministry of Health, Labour and Welfare announced that after the eighth wave of infections subsided, they would leave the decision of wearing masks to individual choice after March 13, 2023 ^39^.

### Real-time version of mobile spatial statistics data

RT-MSS represents the estimated population dynamics for the whole of Japan generated from operational data of the NTT DOCOMO cellular network^40,41^. Population distributions throughout Japan are estimated every ten minutes for each area of the Half Grid Square^42,43^ (an area of approximately 500-m square) according to the attributes of gender, age group (in 5-year increments, from 15 to 79), and place of residence (city). The estimated population of RT-MSS is calculated from the number of mobile phones covered by each base station while taking into account the adoption rate of NTT DOCOMO mobile phones. For more details, please refer to^40^.

To protect user privacy, NTT DOCOMO published guidelines^44^ for generating population data from the operational data of the cellular network. As stated in the guidelines, RT-MSS only provides the fluctuation in population dynamics, not the trajectories of individuals.

The Half Grid Square is a Standard Grid Square demarcation method where the whole area of Japan is divided into square grid areas that do not overlap each other, and is used in the national census. In the Standard Grid Square method, each grid area is designated by a Standard Grid Square Code. For the Half Grid Square, the Standard Grid Square Code comprises nine digits. Hereinafter in this paper, each grid area of the Half Grid Square is referred to as an HGS, and the Standard Grid Square Code of the Half Grid Square is referred to as the HGS Code. For example, the HGS Code for the HGS covering Shinjuku Station in Shinjuku Ward, Tokyo is 533945263.

We define the Kanto area and Kansai area as areas to be analyzed. The Kanto area is an area that is included in Primary Area Partition^43^ 5339 and covers part of Tokyo, Kanagawa, Saitama, Chiba, Ibaraki, and Yamanashi prefectures. The Kansai area is an area that is included in Primary Area Partitions 5135 and 5235 and covers the entire Osaka prefecture and parts of Kyoto, Hyogo, Shiga, Wakayama and Nara prefectures. We used hourly RT-MSS data from 00:00 on January 1, 2019 to 23:00 on March 31, 2023 in the Kanto and Kansai areas. The total population in each HGS was used without attributes. In addition, only HGS with an average population of 100 or more throughout the period were extracted in order to avoid the influence of noise from scarcely populated places. The number of HGS extracted was 15,379 in the Kanto area and 12,699 in the Kansai area.

As a result, $pop_{{\tiny HGS},d,h}$, which is the population of each HGS at each time of each day, is obtained. Here, *d* is the date and *h* is the time. For example, the population of HGS covering Shinjuku Station at 12:00 on January 1, 2019 is $pop_{533945263,20190101,12}$.

### Electronic Disclosure for Investors’ Network (EDINET) Code List

In Japan, Article 24 of the Financial Instruments and Exchange Act requires corporations such as stock-exchange listed corporations to submit securities reports through an electronic disclosure system called EDINET^45^. Data on filed securities reports can be obtained from EDINET. We used the “EDINET Code List” published on EDINET as data to ascertain the number of corporations that exist in each HGS and the amount of their capital. The EDINET Code List contains data on the following items for each corporation that submits annual securities reports: the EDINET code, submitter type, listing category, consolidation status, capital, closing date, submitter name, address, submitter industry, securities code, and submitter corporate number.

First, using the submitter type and submitter industry, we extracted data only for corporations in Japan. Regarding the submitter type, records with the following values were deleted: “foreign corporation/union (other than person obligated to submit securities report, etc.),” “foreign corporation/union,” “Individual (union issuer), (excluding union issuers),” “individuals (non-residents) (excluding union issuers),” and “foreign governments, etc.” For the submitter industry, records with the value of “foreign corporation/union” were deleted. Next, using the Geocoding API of GoogleMapsAPI, we obtained the latitude and longitude of each corporation address and converted them to an HGS Code. We excluded the records where addresses could not be successfully converted to latitudes and longitudes. This yielded the number of filing corporations and the total capital for each HGS.

A total of 6343 corporations were extracted. The number of HGSs where at least one corporation was extracted is 896 in the Kanto area and 454 in the Kansai area. Table 1 gives statistics on the total capital for the above HGSs.

**Table 1:** Statistics of total capital in HGSs with one or more corporations on the EDINET Code List.

	Min	25%	50%	75%	Max
Kanto area	0.00	451.25	3,448.00	18,136.75	17,119,128.00
Kansai area	0.00	491.75	2,320.00	10,951.75	1,734,878.00

The unit of capital is 1,000,000 yen. Capital 0 corresponds to local governments and non-profit organizations.

### Non-negative matrix factorization

Since there are many facilities such as offices, commercial facilities, and residences in each HGS, the population fluctuation in each HGS is a composite of the fluctuations in people engaged in activities such as working, shopping, and watching TV at home. Therefore, in order to analyze the impact of remote work, it is necessary to extract the fluctuations of the working people from those of the population fluctuations in each HGS. We used non-negative matrix factorization (NMF)^35–37^ to decompose the population fluctuations in each HGS. Previous studies showed that the population fluctuation in an area can be expressed as the sum of potential population fluctuation patterns using NMF^46,47^. Here, typical patterns such as “population starts to increase in the morning and starts to decrease in the evening” and “population starts to decrease in the morning and starts to increase in the evening” are extracted as potential fluctuation patterns (daily time-series data). Population fluctuations in each area are expressed as a weighted sum of them.

NMF is a method of expressing a given matrix, *X*, as the product of two matrices whose elements are all non-negative. First, matrix *X* having values of $pop_{HGS,d,h}$ at each HGS, each date, and each time is generated. Matrix *X* has the HGS and date as rows and the time as a column. That is, when the number of HGSs is *a*, the number of dates is *b*, and the number of times is *n*, then *X* is a matrix of $ab \times n$. Here, *a* is 15,379 (HGSs) in the Kanto area, 12,699 (HGSs) in the Kansai area, *b* is 1551 (days), and *n* is 24 (hours). By applying NMF to matrix *X* thus generated, *X* is expressed as the product of two non-negative matrices *W* and *H*. Each row vector of matrix *H* represents a potential population fluctuation pattern during the day. The values in each column of matrix *W* are the weights for each population fluctuation pattern in each HGS on each day.

The number of rows of matrix *H*, i.e., the number of potential population fluctuation patterns to be decomposed, must be determined in advance. In this study, we increased the number of patterns by 1 from 3, observing what kinds of patterns were extracted. When it reached 5, we qualitatively judged that the basic patterns were covered and decided on 5. For applying NMF, decomposition.NMF of Python scikit-learn library version 0.17.1 was used. Parameter “n_components” was set to 5, parameter “max_iter” was set to 100,000, and the others were left as default.

The matrices obtained by applying NMF have degrees of freedom regarding constant multiplication for each population fluctuation pattern. Therefore, matrix *W* and matrix *H* are normalized so that the total value of each population fluctuation pattern becomes 1. Accordingly, the values of matrix *W* (weights) become the population size of each population fluctuation pattern at each HGS on each day.

How to label each pattern extracted by NMF requires knowledge of the lifestyles of the people. In this study, we manually labeled each pattern based on the interpretation of its shape.

### Statistical tests and correlation analysis

As mentioned above, the daily population fluctuation in each HGS is expressed as a weighted sum of five components. Each value of matrix *W* is the weight of each component for each day in each HGS, and since each component is normalized, the weight represents the population size.

By comparing the component of workers, i.e., the weight of the component corresponding to workers, in each HGS before and after the occurrence of an event with a large social impact, it is possible to understand how the number of workers changed in office districts and residential districts. Here, when one day is compared to another day, there is a possibility that they cannot be correctly compared due to the influence of factors such as events and weather. To reduce such effects, weekly averaged values should be compared. In order to focus on the component of working people, the average value for weekdays is used.

In this study, by comparing the states pre- and post-COVID-19 pandemic, we analyze how the number of people working in office and residential areas changed due to the prevalence of remote work. As the post-pandemic period, we use the average value for weekdays of the last week of March 2023 (March 27–31, 2023, hereafter POST-CORONA-WEEK) after the 8th wave of infection subsided. For the pre-pandemic period, we use the weekdays of the same week in 2019 (March 25–29, 2019, hereafter PRE-CORONA-WEEK).

We used the Wilcoxon signed-rank test, which is a paired two-group comparison test, to determine whether or not the component of working people changed significantly in the office districts and residential districts for PRE-CORONA-WEEK and POST-CORONA-WEEK. We assume that the null hypothesis is “there is no change in the component of working people between PRE-CORONA-WEEK and POST-CORONA-WEEK,” and test whether or not it can be rejected at the significance level of 0.01. For the test, stats.wilcoxon of Python Scipy library version 1.6.2 was used. All parameters were default.

For the office districts, we selected HGSs that have one or more corporations listed on the EDINET Code List. For the residential districts, we selected HGSs where there was no corporation listed on the EDINET Code List and the ratio of the components of people at home to the total of all components in PRE-CORONA-WEEK was in the top 25%.

Next, we analyzed the correlation between the changes in the component of working people in office districts and the capital. Here, if the difference pre- and post-pandemic is used as the change in the component of working people, the results will exhibit a trend where the difference is larger in areas where there are more working people. Therefore, we used the “return ratio,” which is the ratio of the component of working people for POST-CORONA-WEEK to PRE-CORONA-WEEK (POST-CORONA-WEEK / PRE-CORONA-WEEK). We calculated the correlation coefficient between the total capital and return ratio in each HGS in office districts. We used DataFrame.corr from the Python pandas library version 1.2.4 for correlation analysis. All parameters were default.

### Ethical statement

Given the nature of the data sources used in this study, no experiments were conducted on humans or human tissue samples. As a result, there were no specific experimental protocols requiring approval from an institutional and/or licensing committee. The study solely involved the analysis of aggregated and anonymized data, ensuring the privacy and confidentiality of the individuals represented in the datasets. Regarding informed consent, since the RT-MSS data does not contain individual-level information, there were no human subjects from whom to obtain informed consent. The data used in this study strictly adheres to NTT DOCOMO’s guidelines^44^ and does not involve the collection of personal data. Overall, this research relied on publicly available data for COVID-19 cases and commercially available RT-MSS data. It conforms to relevant guidelines and regulations, and all analyses were performed using aggregated and anonymized data to ensure the privacy and anonymity of individuals represented in the datasets.

## Results

### Population fluctuation pattern extraction using NMF

**Figure 2:**
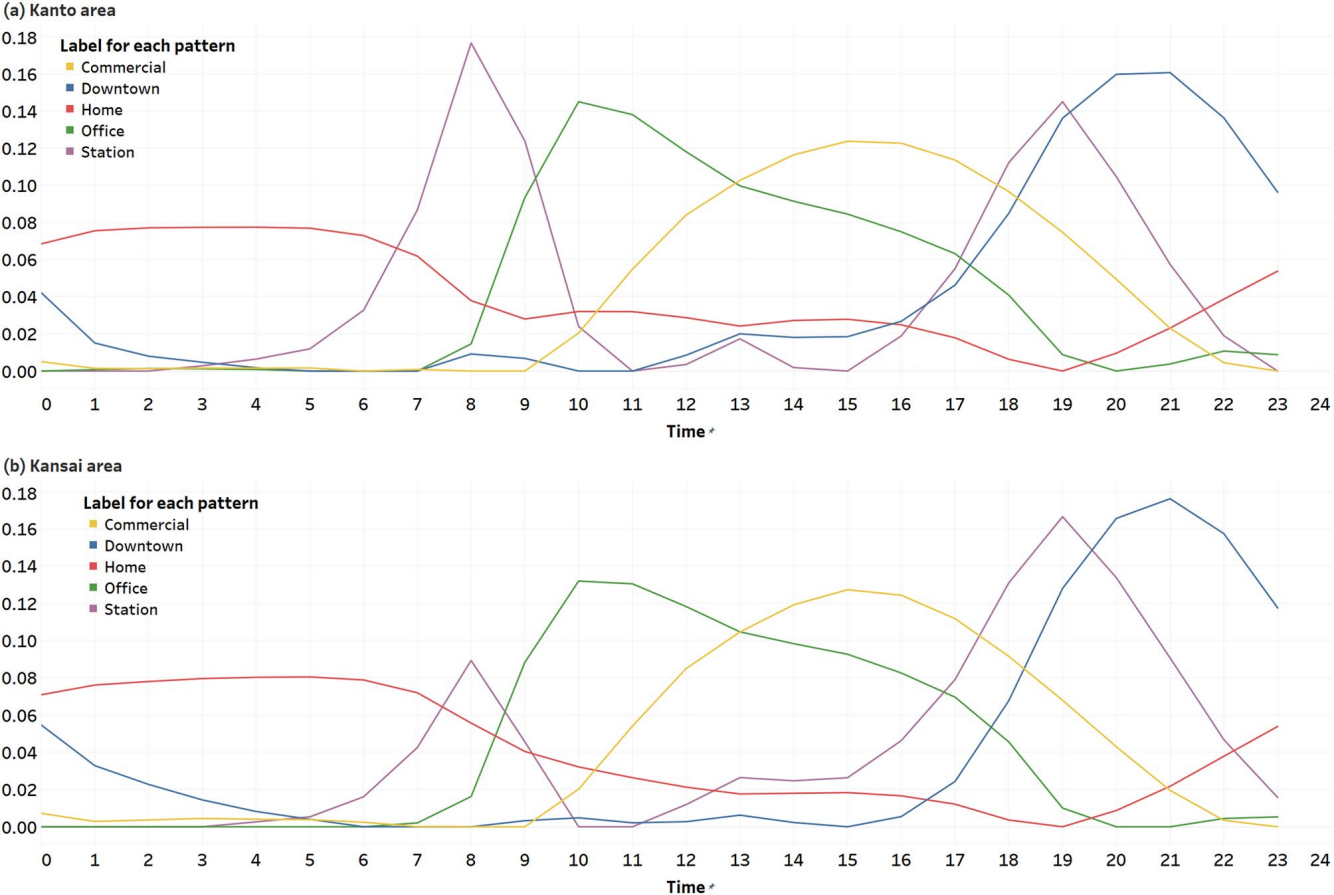
Population fluctuation patterns and their labels. (**a**) Kanto area. (**b**) Kansai area.

Figure 2 shows the (normalized) population fluctuation patterns throughout the day (matrix *H*) in the Kanto and Kansai areas extracted using NMF.

The red pattern appears constantly in large numbers late at night, decreases in the morning, and begins to increase in the evening. This is interpreted as a pattern of people at home and labeled as the “home component.” The purple pattern shows a sharp increase during the morning and evening rush hours and then decreases. This is labeled as the “station component” because it is a pattern due to commuters. The green pattern is then interpreted as a worker pattern and labeled as the “office component” because it increases during the morning rush hour and decreases during the evening rush hour. The orange pattern is labeled as the “commercial component” because it occurs at a later time than the green pattern in terms of both increases and decreases. Finally, the blue pattern increases in the evening and decreases in the middle of the night, so it is considered to be a pattern of people, for example, going to the downtown area after work, and is labeled as the “downtown component.”

Since it is the weight corresponding to each component that is used in the analysis, hereinafter, references to each component refer to the weight. For statistical tests and correlation analyses, we use the office component for those who work and the home component for those who stay at home. Table 2 gives the statistical values of the ratio of the weekday average of each component of each HGS to all components for PRE-CORONA-WEEK.

**Table 2:** Statistical values of the ratio of the weekday average for each component of each HGS to all components on PRE-CORONA-WEEK.

Component	Kanto area						Kansai area					
	Mean	Min	25%	50%	75%	Max	Mean	Min	25%	50%	75%	Max
Home	0.54	0.00	0.49	0.58	0.62	0.82	0.51	0.00	0.47	0.54	0.58	0.74
Station	0.11	0.00	0.10	0.11	0.12	0.41	0.12	0.00	0.10	0.12	0.13	0.41
Office	0.14	0.00	0.08	0.11	0.16	0.66	0.18	0.00	0.11	0.15	0.21	0.77
Commercial	0.10	0.00	0.05	0.08	0.12	0.75	0.10	0.00	0.07	0.09	0.11	0.88
Downtown	0.12	0.00	0.11	0.12	0.14	0.42	0.10	0.00	0.08	0.10	0.12	0.44

**Figure 3:**
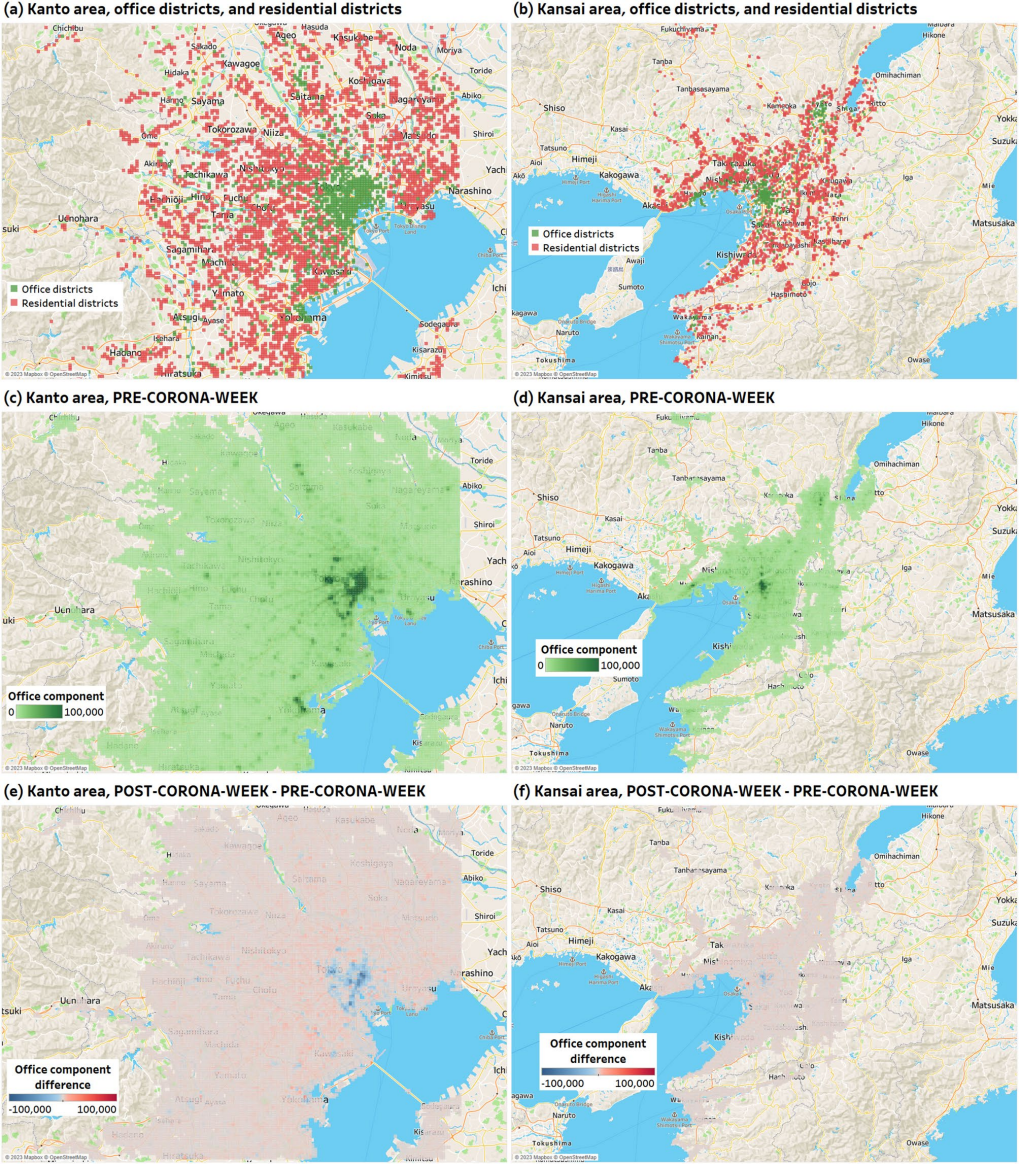
(**a**) and (**b**) Are the distribution of HGS determined to be office districts or residential districts in the Kanto area and the Kansai area, respectively. (**c**) and (**d**) Are heat maps of the office component in the Kanto area and Kansai area on PRE-CORONA-WEEK, respectively. (**e**) and (**f**) Are heat maps of the difference between POST-CORONA-WEEK and PRE-CORONA-WEEK (POST-CORONA-WEEK - PRE-CORONA-WEEK) for office components in the Kanto area and Kansai area, respectively. These figures were all generated using Tableau Desktop Professional Edition version 2021.1.15^48^.

The number of HGS identified as office districts is 896 in the Kanto area and 454 in the Kansai area (equal to the number of HGSs with one or more corporations listed on the EDINET code list). The number of HGS identified as residential districts is 3764 in the Kanto area and 3, 143 in the Kansai area. Figure 3a and b respectively show HGSs identified as office districts and residential districts in the Kanto area and the Kansai area.

Figure 3c and d respectively show heat maps of the office component in the Kanto and Kansai areas for PRE-CORONA-WEEK.

### Changes in office component in office districts and residential districts

Figure 3e and f show heat maps of the difference between POST-CORONA-WEEK and PRE-CORONA-WEEK (POST-CORONA-WEEK - PRE-CORONA-WEEK) for office components in the Kanto area and Kansai area, respectively. For POST-CORONA-WEEK, HGSs with decreased office components compared to those for PRE-CORONA-WEEK are indicated in blue, and HGSs with increased office components are indicated in red.

Table 3 gives statistical values of the difference between POST-CORONA-WEEK and PRE-CORONA-WEEK for office components in the Kanto and Kansai aread. The p-value of the Wilcoxon signed rank test in the office districts is $1.01\cdot 10^{-23}$ for the Kanto area and $6.96\cdot 10^{-11}$ for the Kansai area, both of which are rejected at the significance level of 0.01. The p-value of the Wilcoxon signed rank test in the residential districts is 0.0 (less than the smallest positive number that can be represented by the python floating point number type) for the Kanto area and $7.36\cdot 10^{-144}$ for the Kansai area, both of which are rejected at the significance level of 0.01. Based on the above, we confirm that the number of people working in office districts and residential districts changed significantly in the Kanto and Kansai areas.

**Table 3:** Statistical values of the difference between POST-CORONA-WEEK and PRE-CORONA-WEEK for office components in the Kanto and Kansai areas.

Area	District type	Mean	Min	25%	50%	75%	Max
Kanto	Office	− 3984.72	− 86984.66	− 4693.30	− 465.53	821.94	17116.11
	Residential	763.84	− 2025.13	121.59	555.77	1237.40	6526.24
Kansai	Office	− 1394.88	− 42086.95	− 1887.74	− 414.13	485.27	9955.40
	Residential	297.28	− 2288.43	− 51.49	202.13	549.93	4547.61

**Figure 4:**
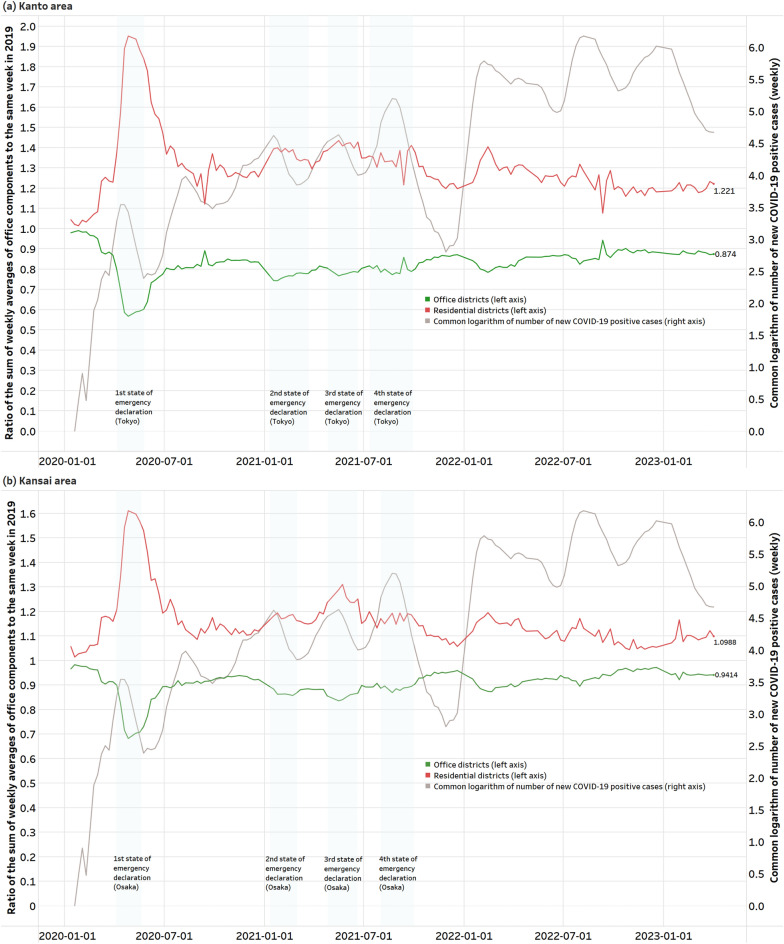
Ratio of the sum of weekly averages of office components (weights) of HGS determined to be office districts or residential districts to the same week in 2019, together with the number of new COVID-19 positive cases (common logarithm). (**a**) Kanto area. (**b**) Kansai area.

Figure 4 shows the ratio of the sum of weekly averages of office components (weights) of HGS determined to be office districts or residential districts to the same week in 2019 in the Kanto and Kansai areas, together with the number of newly confirmed cases of COVID-19. Weeks that include the year-end and New Year holidays, Golden Week holidays, and Obon (August 15), when many people take leave from work in Japan, are excluded. In both areas, from the start of the COVID-19 pandemic to the first declaration of a state of emergency, the office component decreased sharply in office districts, increased rapidly in residential districts, and has not returned to pre-pandemic levels since then.

### Correlation between changes in office component and capital

**Figure 5:**
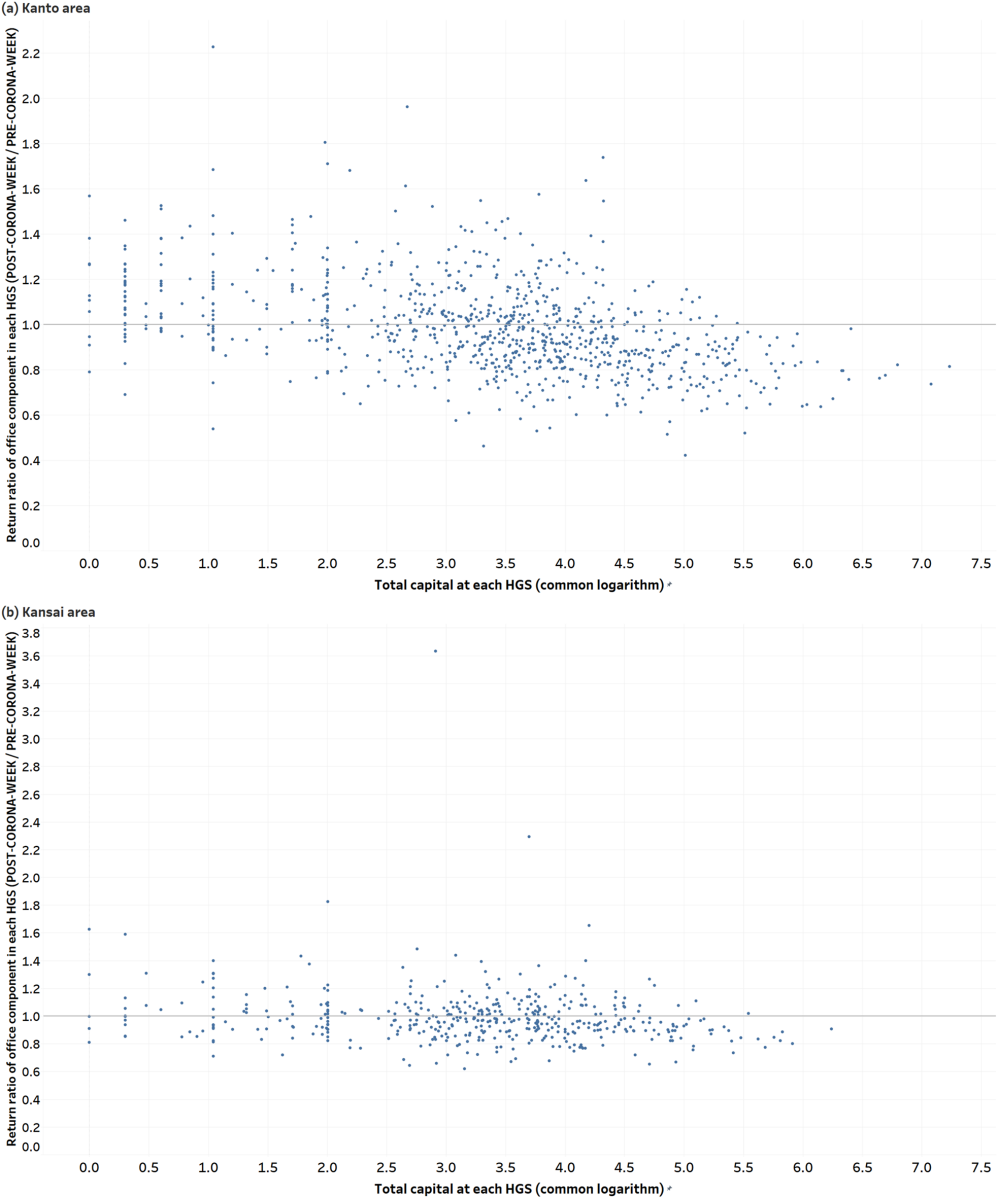
Scatter plot of the return ratio (POST-CORONA-WEEK / PRE-CORONA-WEEK) of the office component and the common logarithm of total capital in each HGS in the office districts of the Kanto and Kansai areas. (**a**) Kanto area. (**b**) Kansai area.

Figure 5 shows a scatter plot of the return ratio (POST-CORONA-WEEK / PRE-CORONA-WEEK) of the office component and the common logarithm of total capital in each HGS in the office districts of the Kanto and Kansai areas. The correlation coefficient is $-0.43$ in the Kanto area and $-\,0.16$ in the Kansai area.

## Discussion

We decomposed the demographic data obtained from the mobile phone network into five population-change patterns using NMF and identified the component of working people (office component). Then, using the office component, we confirmed whether or not the number of people working in office districts and residential districts changed significantly due to the COVID-19 pandemic. The five population change patterns (components) extracted using NMF were almost identical in the Kanto and Kansai areas, which are different metropolitan areas. Also, from Fig. 3, we confirm that the office component, i.e., the component related to workers (the weight) is greater in the city center in both the Kanto and Kansai areas. Based on these, it is considered that life patterns of people can be appropriately extracted using NMF.

Statistical tests on the difference in office components between PRE-CORONA-WEEK and POST-CORONA-WEEK confirmed that the office components changed significantly pre- and post-COVID-19 pandemic in both office and residential districts. Based on Fig. 4, it is thought that the number of working people decreased in office districts and increased in residential districts. The change in the office component began around March 2020, when news regarding COVID-19 began to appear in Japan, and accelerated with the first state of emergency declaration. Since then, it has gradually returned to a pre-pandemic level, although repeated peaks and troughs have occurred due to the spread of infection and the declaration of a state of emergency. With the exception of the 2nd wave and the 8th wave in the Kanto area, when the infection spreads, the number of people working in office districts has decreased and the number of people working in residential districts has increased. However as time passed since the start of the pandemic, this fluctuation has become less sensitive to the spread of infection. In the Kanto area, there has been no change since the 7th wave avated, and there is no response to the 8th wave. Even in the Kansai area, after the 7th wave abated, there was no change in the level after a slight response to the 8th wave. During the second wave of infection, the momentum to return to a pre-pabdemic level weakened after the end of the first wave, but the number of people working in office districts did not decrease. This is thought to be due to the fact that the government did not declare a state of emergency and implemented a policy called the GoTo campaign to promote travel and dining out. As a result, people’s sense of crisis regarding the spread of infection did not increase significantly. In the end, neither area has returned to pre-pandemic levels. This suggests that remote work, which has become prevalent due to the COVID-19 pandemic, has taken root to some extent, but the degree differs between the Kanto and Kansai areas. In the Kanto area, compared to 2019 before the COVID-19 pandemic, the office component in office districts decreased by approximately 13%, while that in residential districts increased by approximately 22%. On the other hand, in the Kansai area, office components decreased by approximately 6% in office districts and increased by approximately 10% in residential districts. Here, the amount of increase and decrease in the office district and the residential district do not match, but they do not necessarily balance because there are movements outside the area.

In Fig. 4, apart from the overall trend, the ratio changes sharply in the second week of September every year in the Kanto area. This is thought to be because Typhoon Faxay, which caused extensive damage ^49^, landed in the Kanto area in the second week of September 2019, and many people refrained from going out.

We found a moderate negative correlation in the Kanto area between the total amount of corporate capital in each HGS and the return ratio of office components pre- and post-COVID-19 pandemic. At an individual corporation level, factors such as whether or not the corporation introduced remote work and whether or not the business type is suitable for remote work are considered to have a large impact. However, as a general trend, it was confirmed that the number of working people decreased in HGSs where many corporations with large capital gather. This is in line with the trend obtained from past questionnaire surveys^27^ that the implementation rate of remote work is higher in large corporations than in small and medium-sized corporations. For HGS where the capital size exceeds a certain line ($10^{5.5}$), the return rate does not exceed 1.0. On the other hand, no correlation could be confirmed in the Kansai area. As Fig. 4 shows, the adoption of remote work is less advanced in the Kansai area than in the Kanto area. Therefore, it is considered that there is insufficient difference to make the correlation significant pre- and post-COVID-19 pandemic.

This study has made it possible to quantitatively assess the state of prevalence of remote work on an area-by-area basis, going beyond the previous limitations of relying on surveys to understand overall trends. The results of this study are considered valuable data for decision-making in urban development, infrastructure investments, disaster preparedness, and other areas. The significant changes observed in office components in both office districts and residential districts may have implications for various aspects such as commuter traffic volume, train occupancy rates during rush hours, electricity usage, demand for dining establishments, network traffic volume, and office vacancy rates. For instance, the increase in people working from home could lead to a decrease in electricity usage and dining establishments in office districts while simultaneously increasing the demand in residential districts. Moreover, as individuals conduct online meetings from their homes, there may be an increase in network traffic, not only for downloading but also uploading, in residential districts that previously had lower daytime traffic. Additionally, areas with a higher concentration of large-capital companies tend to have lower return rates of office components, suggesting that central business districts with numerous large enterprises may experience an increase in office vacancy rates. Furthermore, as can be seen from Figs. 3 and 5, some HGSs identified as office districts in this study have seen an increase in office components compared to the pre-COVID-19 period, while others have witnessed a decrease, with varying return rates. This data can provide essential insights for informed decision-making in various aspects of urban development and emergency planning.

Up to this point, we have attributed the changes in office components to the impact of remote work. However, it’s important to acknowledge the possibility that some companies, much like schools that were closed, may have suspended their regular operations during the COVID-19 pandemic. Especially during the state of emergency, while not legally binding, requests were made for non-essential workers to refrain from going to the office and for commercial facilities to suspend their operations. Therefore, not all changes in office components can be solely attributed to remote work. Nevertheless, even after the state of emergency ended, office components did not return to their pre-pandemic levels and continued to fluctuate in response to waves of infection. This suggests that, at least these changes are due to remote work. Especially during the sixth wave when the Omicron variant emerged, and infections rapidly increased, significant changes in office components were observed, even in the absence of a state of emergency. This may indicate that remote work has become firmly established, allowing for flexible adjustments in work arrangements in response to the evolving infection situation.

Furthermore, the results of the correlation analysis between area capital size and return ratios suggests that such flexibility is more likely in areas with larger capital sizes. As mentioned in the introduction, remote work offers various advantages and has become an important factor for job seekers when choosing their place of employment, especially among younger generations^4,15^. In Japan’s job market, there is a significant difference in job opening-to-applicants ratio between small and medium-sized enterprises (with capital of less than 300 million yen and fewer than 300 employees) and large enterprises^50^. The job opening-to-applicants ratio for large enterprises generally falls below 1.0, while for small and medium-sized enterprises, it can range from around 4 times to as much as 10 times, depending on the year. If remote work continues to be more prevalent at larger companies, the number of job seekers who aspire to work at large companies may increase further in the future. Although the adoption rate of remote work is higher in larger companies, even in smaller companies that have implemented remote work, the proportion of those experiencing the benefits of its implementation is equal to or higher than that of larger companies^51^. Therefore, it is considered desirable for small and medium-sized enterprises to actively transition to remote work for roles where it can be applied.

Finally, we discuss the limitations of this study and future work. As mentioned earlier, it’s important to note that not all changes in office components during a state of emergency can be attributed solely to remote work. Next, in this study, using the EDINET code list, HSGs with one or more Japanese domestic corporations submitting annual securities reports were selected as office districts. However, only a small portion of the approximately 3.67 million Japanese corporations^52^ has filed annual securities reports. Even for corporations submitting annual securities reports, their offices other than the head office have not been considered. There may be cases where a corporate headquarters exists in a residential area. In that case, even if the number of people in the office is decreasing due to the prevalence of remote work, it may be offset by the increase in residential areas. By using a broader and more detailed database of corporate offices, it may be possible to clarify the relationship with the prevalence of remote work, considering not only the capital size but also other factors such as the number of corporations, the number of employees, and the type of business. Furthermore, while this study defined office districts and residential districts for analysis, to make practical use of the results, it is necessary to focus on each HGS and delve into the finer details of changes in the spatial distribution of working people, including the differences in characteristics between HGS where the office component has increased and those where it has decreased. In addition, in this study, we focused on the office component to clarify the prevalence of remote work. Future work may be able to clarify more broadly how life patterns of people have changed due to the COVID-19 pandemic by also focusing on the commercial and downtown components.

## Data Availability

RT-MSS data are commercially available from DOCOMO Insight Marketing, Inc. EDINET Code List is available at https://disclosure2.edinet-fsa.go.jp/.
